# Whole Genome Sequencing and Biocontrol Potential of *Streptomyces luteireticuli* ASG80 Against *Phytophthora* Diseases

**DOI:** 10.3390/microorganisms12112255

**Published:** 2024-11-07

**Authors:** Gang Xu, Weihuai Wu, Liqian Zhu, Yanqiong Liang, Minli Liang, Shibei Tan, Helong Chen, Xing Huang, Chunping He, Ying Lu, Kexian Yi, Xiang Ma

**Affiliations:** 1 Pathogenesis and Control of Pathogenic Microorganisms Research Team, School of Life and Health Sciences, Hainan Province Key Laboratory of One Health, Collaborative Innovation Center of One Health, Hainan University, Haikou 570228, China; 2 Hainan Key Laboratory for Monitoring and Control of Tropical Agricultural Pests, Environment and Plant Protection Institute, Chinese Academy of Tropical Agricultural Sciences, Haikou 571101, China; weihuaiwu2002@163.com (W.W.);; 3 College of Plant Science and Technology, Huazhong Agricultural University, Wuhan 430070, China; 4 Sanya Research Insatitute, Chinese Academy of Tropical Agricultural Sciences, Sanya 572025, China

**Keywords:** *Phytophthora*, *Streptomyces*, antimicrobial activity, biocontrol agent, whole genome sequencing

## Abstract

*Phytophthora*-induced crop diseases, commonly known as “plant plagues”, pose a significant threat to global food security. In this study, strain ASG80 was isolated from sisal roots and demonstrated a broad-spectrum antagonistic activity against several *Phytophthora* species and fungal pathogens. Strain ASG80 was identified as *Streptomyces luteireticuli* via phylogenetic analysis, digital DNA–DNA hybridization (dDDH), and average nucleotide identity (ANI). Whole-genome sequencing identified 40 biosynthetic gene clusters (BGCs) related to secondary metabolite production, including antimicrobial compounds. Strain ASG80 extract exhibited broad-spectrum inhibitory activity against *Phytophthora nicotianae*, *P. vignae*, *P. cinnamomi*, and *P. sojae*. Pot experiments showed that strain ASG80 extract significantly reduced sisal zebra disease incidence, with an efficacy comparable to the fungicide metalaxyl. These findings suggest that strain ASG80 is a promising biocontrol agent with substantial potential for managing Phytophthora-related diseases in agriculture.

## 1. Introduction

*Phytophthora*, a genus of oomycetes, is widely recognized for causing severe plant diseases that have a substantial impact on global agriculture [1]. This genus, often referred to as the “Plant Destroyer”, currently encompasses over 400 described species and subspecies (www.mycobank.org, accessed on 15 August 2024). In recent years, the incidence of diseases caused by *Phytophthora* has been increasing, a trend attributed to climate change and intensified agricultural activities [2]. The pathogens within this genus exhibit varying degrees of virulence and host specificity, resulting in a diverse range of effects on crops. For example, *Phytophthora infestans*, the primary pathogen responsible for potato late blight, instigated the Irish potato famine in the 19th century and continues to cause an estimated annual economic loss of over USD 6 billion globally [3]. *P. ramorum*, the causal agent of sudden oak death, has significantly disrupted forest ecosystems throughout North America [4]. *P. cinnamomi*, a major pathogen of root rot in avocados, poses a substantial threat to the global avocado industry [5]. Additionally, *P. capsici* infects a range of economically important vegetables, causing root, stem, leaf, and fruit rot. This pathogen has a broad host range exceeding 50 cultivated plant species, leading to considerable economic losses [6,7]. *P. sojae* is responsible for root and stem rot in soybeans, causing estimated global losses ranging from USD 1 to USD 2 billion annually [8]. *P. nicotianae* has adversely affected numerous economically significant crops, with tobacco production losses in North Carolina, USA, alone exceeding USD 30 million annually [9]. Lastly, *P. palmivora* is a major pathogen affecting tropical crops, most notably causing bud rot in African oil palms, a disease ranked among the most destructive in this category [10]. In efforts to manage *Phytophthora*-induced diseases, researchers have investigated a range of strategies. Chemical control remains among the most widely used methods; however, prolonged application of single-target pesticides may result in resistance and residue accumulation, raising concerns regarding food safety and human health. Consequently, biological control has become an essential alternative to chemical fungicides in agricultural practices [11].

Biological control, as an environmentally sustainable approach to disease management, has gained substantial attention in recent years. Biological control involves the use of biological agents, such as beneficial microorganisms or their metabolites, to suppress or control plant pathogens, offering multiple advantages. Firstly, biocontrol is environmentally friendly. In contrast to chemical pesticides, biological control agents generally exert a lower environmental impact, thereby reducing the risks of soil and water pollution [12,13]. Secondly, biocontrol effectively addresses challenges associated with pathogen resistance. Long-term use of chemical pesticides often results in the development of pathogen resistance. In contrast, biocontrol strategies employ multiple mechanisms—such as competition, antagonism, and induced resistance—which collectively reduce the likelihood of resistance evolution [14,15,16,17]. Additionally, biocontrol is safe and sustainable. Many biocontrol agents are safe for humans and animals, and they can be integrated with other agricultural practices as part of an integrated disease management strategy [18,19]. Lastly, biocontrol agents often offer long-term disease control by persisting in the soil and continuing to combat pathogens over extended periods [19,20].

Currently, agricultural biocontrol methods include microbial control, natural enemies, insect pathogens, plant extracts, and insect pheromones. Microbial control has attracted significant attention in plant disease management. Microbial antagonists, which include beneficial bacteria, fungi, and viruses, are utilized to control plant diseases [21]. Certain secondary metabolites produced by these microorganisms have been proven effective in managing plant pathogens. For instance, secondary metabolites from *Trichoderma* spp. demonstrate strong antimicrobial activity against *Lasiodiplodia theobromae*, *Xanthomonas campestris*, and *Meloidogyne incognita* [22,23]. Additionally, metabolites produced by *Bacillus subtilis* JF-4, *B. amylum* JF-5 and *Pseudomonas aeruginosa* have shown efficacy in controlling banana anthracnose [24,25].

Research has shown that secondary metabolites from *Streptomyces* species exhibit broad-spectrum antibacterial, antiviral, and anthelmintic properties, highlighting the potential of *Streptomyces* as biocontrol agents [26,27,28]. For instance, Park et al. isolated *Streptomyces roseoflavus* LS-A24, which produces staurosporine in its fermentation broth, exhibiting inhibitory activity against *Phytophthora capsici*, the pathogen responsible for pepper blight [29]. Sun et al. reported an exopolysaccharide, EPS66A, from *Streptomyces* sp. HL-66, which induces resistance in tobacco against tobacco mosaic virus [30]. Moreover, Wang et al. isolated novel macrocyclic lactones from *S. avermitilis* NEAU1069 that showed strong acaricidal and anthelmintic activities [31]. Arasu et al. isolated a novel polyketide compound from *Streptomyces* sp. AP-123, which displayed potent broad-spectrum antibacterial, antifungal, and cytotoxic activities, comparable to erythromycin [32]. Therefore, the utilization of *Streptomyces* species for plant disease control emerges as a promising strategy.

In this study, we isolated and screened an antimicrobial strain, ASG80, from the roots of *sisal*. Whole-genome sequencing was performed on strain ASG80 to predict potential biocontrol mechanisms, likely linked to secondary metabolites with antifungal properties. Subsequently, the strain ASG80 extract was evaluated against plant pathogens, demonstrating a broad inhibitory spectrum against *Phytophthora* species and indicating its potential as a biocontrol agent. Additionally, pot trials were conducted to confirm its efficacy against sisal zebra disease, providing strong evidence for its application in the management of *Phytophthora*-related diseases. These results suggest that strain ASG80 represents a valuable microbial resource for the future control of Phytophthora plant diseases.

## 2. Materials and Methods

### 2.1. Isolation of Streptomyces

Soil samples were collected from the root zone of sisal in Haikou, Hainan Province, China. The samples were stored in sealed bags at 4 °C until further processing. A 10 g portion of the root sample was ground using a sterile mortar and pestle, transferred to an Erlenmeyer flask containing 90 mL of sterile water, and agitated for 20 min. A 1 mL aliquot of the suspension was serially diluted up to 10^4^-fold, and the 10^−2^, 10^−3^, and 10^−4^ dilutions were spread onto Gauze’s No. 1 solid medium plates (soluble starch, 20.0 g; KNO_3_, 1.0 g; K_2_HPO_4_, 0.5 g; MgSO_4_·7H_2_O, 0.5 g; NaCl, 0.5 g; FeSO_4_·7H_2_O, 0.01 g; agar, 20.0 g; pH 7.4–7.6), containing 1 µg/mL nystatin, nalidixic acid, and potassium dichromate as selective agents. Plates were incubated at 28 °C, with each dilution plated in triplicate. Colonies were subsequently selected from the plates, and the strain was isolated and purified on fresh ISP2 solid medium (yeast extract, 4.0 g; malt extract, 10.0 g; glucose, 4.0 g; trace salt solution, 1.0 mL; agar, 20.0 g; distilled water, 1000 mL; pH 7.2). The trace salt solution consisted of FeSO_4_·7H_2_O, 0.1 g; MnCl_2_·4H_2_O, 0.1 g; ZnSO_4_·7H_2_O, 0.1 g; distilled water, 1000 mL. Plates were autoclaved at 121 °C for 20 min. The purified strain was preserved in 20% glycerol at −80 °C for long-term storage.

Nystatin and nalidixic acid were purchased from Shanghai Lin’en Technology Development Co., Ltd., (Shanghai, China). All other chemicals were purchased from Xilong Scientific Technology Co., Ltd., (Guangzhou, China).

### 2.2. Screening of Streptomyces Strains with Anti-Phytophthora Activity and Antifungal Spectrum Assays

The antagonistic activity of *Streptomyces* strains against *Phytophthora nicotianae* was assessed using the dual-culture plate assay. A mycelial plug of *P. nicotianae* was placed at the center of a PDA plate, and each *Streptomyces* strain was inoculated at four equidistant points approximately 2 cm from the center. Plates inoculated with *P. nicotianae* alone served as the control [33]. The plates were incubated at 28 °C until the fungal growth in the control group completely covered the plate. Each treatment was conducted in triplicate. The fungal colony diameter was measured using the cross-measurement method, and the inhibition rate of each *Streptomyces* strain was calculated. The inhibition rate (%) was determined using the formula: inhibition rate (%) = [(colony diameter of control − colony diameter of treatment)/(colony diameter of control − diameter of fungal plug)] × 100%.

The strain exhibiting the highest antimicrobial activity was selected for further antifungal spectrum assays. The evaluated pathogens included plant pathogenic oomycetes: *Phytophthora nicotianae*, *P. cinnamomi*, *P. palmivora*, *P. capsici*, *P. vignae*, *P. colocasiae*, *P. sojae*, and *P. melonis*. Additionally, several plant pathogenic fungi were tested, including *Colletotrichum gloeosporioides*, *Fusarium graminearum*, *Magnaporthe oryzae*, *F*. *oxysporum*, and *Pestalotiopsis palmarum*. For each pathogen, a 5 mm mycelial plug was placed at the center of a PDA plate (90 mm diameter), and strain ASG80 was inoculated at two positions, 2 cm above and below the plug. Plates inoculated only with the pathogen served as controls. The plates were incubated at 28 °C until fungal growth in the control group fully covered the plate. Each treatment was conducted in triplicate. The colony diameter was measured using the cross-measurement method, and the inhibition rate of strain ASG80 against each pathogen was calculated as follows: inhibition rate (%) = [(control colony diameter − treatment colony diameter)/(control colony diameter − pathogen plug diameter)] × 100%.

### 2.3. Genome Assembly and Annotation

Sample preparation and genome sequencing were performed as previously described by Wang et al. The ASG80 sample was sequenced at Biomarker Technologies (Beijing, China) using single-molecule real-time sequencing on the PacBio platform. Genome assembly and functional annotation followed the methods outlined by Wang et al. [34]. The genome of *Streptomyces luteireticuli* ASG80 was annotated using Prodigal (v2.6.3) [35]. rRNA and tRNA genes were identified with Infernal (v1.1.3) [36] and tRNAscan-SE (v2.0) [37], respectively. Functional annotation was conducted using BLASTx against multiple databases, including the NCBI non-redundant protein database (Nr), gene ontology (GO), Kyoto Encyclopedia of Genes and Genomes (KEGG), Clusters of Orthologous Groups (COG), and Swiss-Prot. Gene clusters involved in secondary metabolite synthesis were identified using antiSMASH 7.1.0 [38].

The 16S rRNA gene sequence of strain ASG80 was extracted using Barrnap (version 0.9). This sequence, along with 16S rRNA sequences from closely related *Streptomyces* strains downloaded from the EZBioCloud database, was used to construct a preliminary 16S rRNA-based phylogenetic tree to clarify the taxonomic position of strain ASG80 and its relationships with related strains. A phylogenetic tree was generated using Molecular Evolutionary Genetic Analysis software (version 7.0, MEGA, Auckland, New Zealand). Based on the results of the 16S rRNA phylogenetic tree, genomes of closely related species were retrieved from the GenBank database. A whole-genome phylogenetic tree was then constructed online using the Type Strain Genome Server (https://tygs.dsmz.de/, accessed on 16 July 2024), and digital DNA–DNA hybridization (dDDH) values were calculated [38]. Additionally, whole-genome comparisons between strain ASG80 and its closely related species were conducted using JSpeciesWS (http://jspecies.ribohost.com/jspeciesws/#analyse, accessed on 16 July 2024) to obtain average nucleotide identity (ANI) values. The taxonomic classification of the isolated *Streptomyces* strain was confirmed based on the dDDH and ANI values.

### 2.4. Comparative Genomic Analysis

To analyze the phylogenetic relationships and genomic similarities, three strains most closely related to strain ASG80 were identified based on phylogenetic analysis, ANI values, and dDDH value comparisons. The selected strains were *Streptomyces luteireticuli* JCM 4788 (GenBank Accession No.: GCA_039521205.1), *S. thioluteus* JCM 4087 (GCA_039535865.1), and *S. caatingaensis* CMAA 1322 (GCA_001187435.1). A comparative genomic analysis was performed using OrthoMCL (v2.0) to classify protein families from strain ASG80 and the reference genomes [39]. A Venn or petal diagram was constructed to illustrate the gene family statistics. A BLAST analysis was executed to compare the protein sequences of strain ASG80 with those of the reference genomes, allowing the identification of homologous genes. Syntenic relationships at the nucleotide level were established based on their respective genomic loci. Pairwise synteny plots between the ASG80 genome and each reference genome were generated using MCScanX software (https://github.com/wyp1125/MCScanX, accessed on 23 July 2020) [40].

### 2.5. Preparation of Ethyl Acetate Extract

Strain ASG80 was first activated on ISP2 solid medium plates and subsequently inoculated into ISP2 liquid medium. The culture was incubated at 28 °C with shaking at 180 rpm for 3 days. A 1% (*v*/*v*) inoculum was then transferred to a soybean meal infusion medium (containing sucrose, 10.0 g; peptone, 2.0 g; soluble starch, 5.0 g; yeast extract, 2.0 g; NaCl, 2.0 g; K_2_HPO_4_, 0.5 g; MgSO_4_·7H_2_O, 0.5 g; CaCO_3_, 1.0 g; soybean meal, 20.0 g; distilled water, 1000 mL; pH 7.2). The medium was sterilized by autoclaving at 121 °C for 20 min. The culture was then incubated at 28 °C and 180 rpm for 8 days to obtain the fermentation broth. The fermentation broth was extracted three times with an equal volume of ethyl acetate. The combined ethyl acetate extracts were concentrated to dryness under reduced pressure at 45 °C to yield the crude extract, representing the fermentation product of strain ASG80.

### 2.6. Inhibition Rate of Mycelial Growth by ASG80 Extract

The inhibitory activity of the ASG80 extract against mycelial growth of plant pathogens was evaluated using the growth rate method. The extract was dissolved in DMSO at a concentration of 10.0 mg/mL and added to PDA or V8 medium at 50–60 °C. A series of PDA or V8 plates with varying concentrations of the active compound were prepared using two-fold serial dilutions, resulting in final concentrations of 0.125 μg/mL, 0.25 μg/mL, 0.5 μg/mL, 1 μg/mL, 2 μg/mL, 4 μg/mL, and 8 μg/mL. Plates with an equal volume of DMSO served as controls. Each plant pathogen was inoculated at the center of the plates, which were then incubated at 28 °C until the mycelia in the control group reached the plate edge. The average vertical diameter of the colonies was measured. Each treatment was performed in triplicate. The median effective concentration (EC_50_) values for mycelial growth inhibition were calculated to determine the extract’s efficacy against plant pathogens [41].

### 2.7. Pot Experiment for Disease Control with ASG80 Extract

The inoculum of *Phytophthora nicotianae* was prepared following established methods. Millet grains were boiled until approximately two-thirds of the husks cracked, then filtered through gauze and air-dried to approximately 40% moisture content. The grains were placed in Erlenmeyer flasks and sterilized at 121 °C for 20 min. *P. nicotianae* was cultured on V8 medium for 4 days, after which five 5 mm mycelial plugs were inoculated onto the sterilized millet medium. The cultures were then incubated at 28 °C for 14 days [42].

The test soil, obtained from Huizhou Bida Landscape Materials Co., Ltd., was mixed with *P. nicotianae*-infested millet to create infected soil. Each pot was filled with 1 kg of soil, containing 4 g of inoculated millet. Uniform 0.5-leaf-stage sisal seedlings were transplanted into the treated soil and immediately received the designated treatments. Five treatment groups were established: CK (no *P. nicotianae*, sterile water), Pn (*P. nicotianae*, sterile water), T-1 (*P. nicotianae*, ASG80 extract at a 1:1000 dilution), T-2 (*P. nicotianae*, ASG80 extract at a 1:2000 dilution), and Me-2 (*P. nicotianae*, metalaxyl at a 1:2000 dilution). For each treatment, 20 mL of solution was applied to each pot for root drenching. Disease incidence and severity were assessed on individual plants 30 days after transplanting, with disease index and control efficacy subsequently calculated.

## 3. Results

### 3.1. Screening of Streptomyces

**Figure 1:**
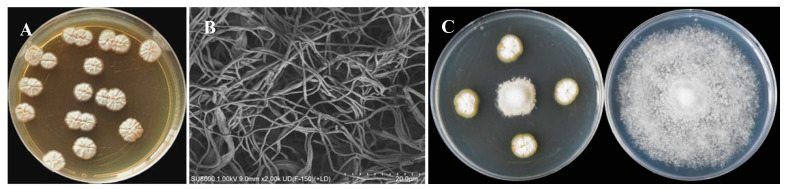
Isolation of strain ASG80 with strong antifungal activity against *Phytophthora nicotianae*. (**A**) The colony morphology of strain ASG80. (**B**) Morphological characteristics of aerial mycelia of strain ASG80 using SEM. (**C**) Strain ASG80 extracts inhibiting mycelial growth of *P*. *nicotianae*.

A total of 28 strains of *Streptomyces* with antagonistic activity against *Phytophthora nicotianae* were isolated from the roots of sisal. Notably, strain ASG80 exhibited the strongest antibacterial activity, achieving a growth inhibition rate of 80.51 ± 0.16% (Figure 1A). On ISP2 solid medium, strain ASG80 formed light yellow substrate mycelium, while the aerial mycelium appeared white (Figure 1B). Scanning electron microscopy (SEM) (SU8600, HITACHI, Tokyo, Japan) revealed that the aerial mycelium exhibited a filamentous structure (Figure 1C).

### 3.2. Antifungal Spectrum of Strain ASG80

The antifungal spectrum of strain ASG80 was evaluated using a standard spot inoculation method. Strain ASG80 exhibited broad-spectrum antifungal activity, with varying degrees of inhibition observed against all 12 tested pathogens. Notably, it showed strong inhibitory effects against oomycetes such as *Phytophthora cinnamomi*, *P. palmivora*, *P. capsici*, *P. vignae*, *P. colocasiae*, *P. sojae*, and *P. melonis*, achieving a maximum inhibition rate of 88.43 ± 0.31. The inhibition rate against *Magnaporthe oryzae* was 85.87 ± 0. In contrast, the inhibitory effects on *Colletotrichum gloeosporioides*, *Fusarium graminearum*, Magnaporthe *oryzae*, *F. oxysporum*, and *Pestalotiopsis palmarum* were relatively lower (Table 1; Supplementary Figure S1).

### 3.3. Complete Genome Sequence and Biosynthesis-Related Gene Clusters of Strain ASG80

**Figure 2:**
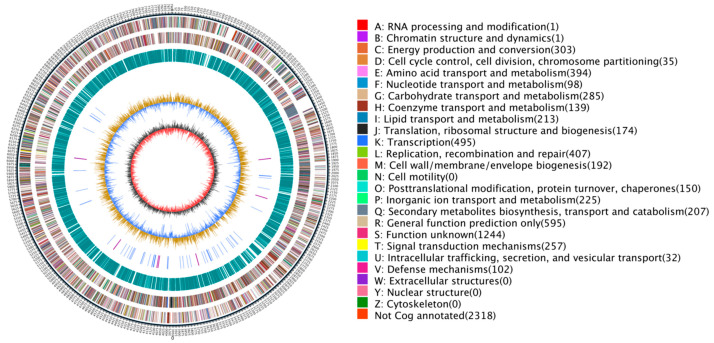
Genome map of strain ASG80: The outermost circle represents the genome size. The second and third circles depict genes on the positive and negative strands of the genome, with different colors indicating various COG functional classifications. The fourth circle displays repeat sequences, while the fifth indicates tRNA (blue) and rRNA (purple). The sixth circle represents GC content, and the innermost circle shows the GC skew. The letters A–Z correspond to the functional classification of CDS genes in the chromosome.

The complete genome of strain ASG80 was sequenced using single-molecule real-time (SMRT) sequencing on the PacBio platform and assembled de novo into four contigs, consisting of one chromosomal DNA (accession number: CP167927) and three plasmid DNAs (accession numbers: CP167926-CP167930). The total genome length of ASG80 is 8,724,805 bp, with a GC content of 71.77%, and it encodes 7867 genes (Figure 2). Coding sequences constitute 87.01% of the genome, with an average gene length of 964 bp. Structural analysis revealed that the ASG80 genome contains 21 rRNAs, 80 tRNAs, four CRISPR arrays, four genomic islands, three prophages, and five operons. Functional annotation of the 7867 genes was performed using eggNOG, GO, KEGG, Nr, Swiss-Prot, TrEMBL, and Pfam databases, resulting in the annotation of 7549 genes (97.50%) across all databases (Table 2).

The antiSMASH v7.1.0 analysis predicted 40 biosynthetic gene clusters for secondary metabolites within the ASG80 genome (Table 3). Most bioactive compounds produced by *Streptomyces*, such as insecticides, antibiotics, and anticancer agents, are synthesized through polyketide synthase (PKS) and non-ribosomal peptide synthase (NRPS) pathways. The strain ASG80 genome contains 25 gene clusters related to PKS and NRPS pathways, accounting for 62.5% of all gene clusters. These include 13 T1PKS, 1 T2PKS, 14 NRPS, and 6 NRPS-like clusters. Additional gene clusters identified include nine terpene, two lasso peptides, one NAPAA, three phenazine, one thioamide-NRP, one butyrolactone, one CDPS, one furan, three RiPP-like, one thiopeptide, two NI-siderophore, and one indole cluster.

Among the 40 gene clusters encoded in the strain ASG80 genome, four clusters (Clusters 1, 8, 11, and 12) showed 100% similarity to known gene clusters. An additional three clusters (Clusters 16, 38, and 39) exhibited over 80% similarity, indicating that strain ASG80 likely has the potential to produce these secondary metabolites. Meanwhile, 27 clusters displayed similarities ranging from 4% to 76%, suggesting the potential for strain ASG80 to produce known metabolites or their structural analogs. The remaining six clusters showed no significant matches, suggesting they may represent novel gene clusters.

**Figure 3:**
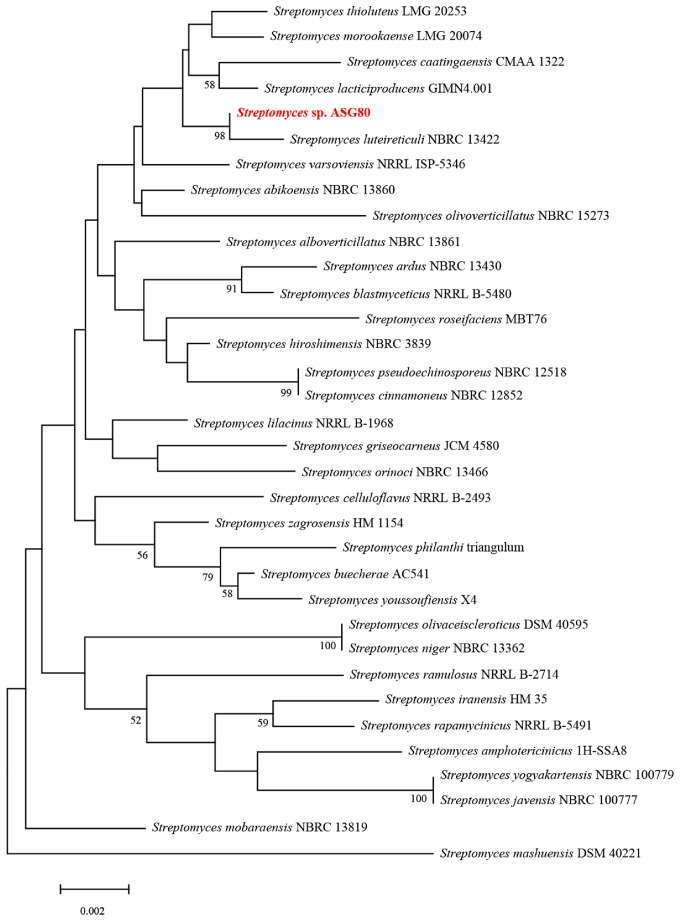
Phylogenetic tree of strain ASG80 based on 16S rRNA gene sequence analysis. The bootstrap values (%) at the branches were calculated from 1000 replications.

**Figure 4:**
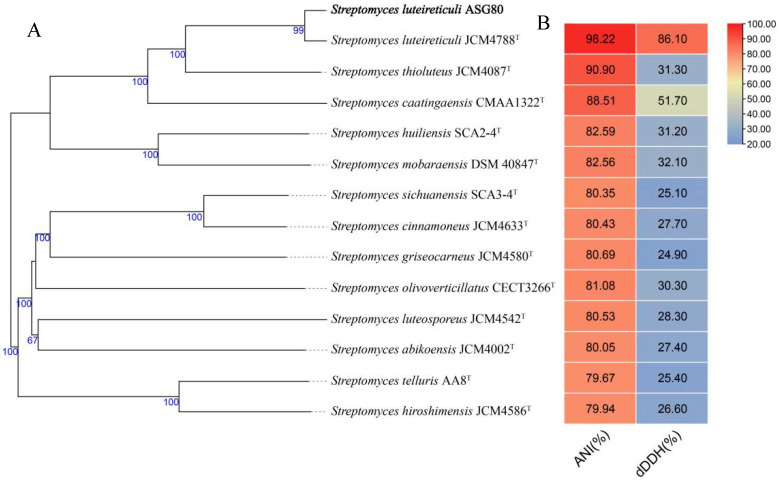
Analysis of strain ASG80 and related *Streptomyces* species. (**A**) A phylogenetic tree was constructed using the Type Strain Genome Server to analyze the whole genome sequences. (**B**) Heatmaps were generated to visualize the calculated average nucleotide identity (ANI) values and digital DNA–DNA hybridization (dDDH) values. ^T^: Type strain.

Phylogenetic and homology analysis using the 16S rDNA sequence revealed that strain ASG80 shares the highest similarity (99.86%) with *Streptomyces luteireticuli* NBRC 13422^T^, clustering closely with this species (Figure 3). A phylogenetic tree was constructed based on the whole-genome sequences of ASG80, *S. luteireticuli* JCM 4788^T^, *S. thioluteus* JCM 4087^T^, *S. caatingaensis* CMAA 1322^T^, *S. huiliensis* SCA2-4^T^, *S. mobaraensis* DSM 40847^T^, *S. cinnamoneus* JCM 4633^T^, *S. sichuanensis* SCA3-4^T^, *S. griseocarneus* JCM 4580^T^, *S. olivoverticillatus* CECT 3266^T^, *S. hiroshimensis* JCM 4586^T^, *S. telluris* AA8^T^, *S. abikoensis* JCM 4002^T^, and *S. luteosporeus* JCM 4542^T^ (Figure 4A). The results indicate that strain ASG80 is most closely related to *S. luteireticuli* JCM 4788, while it is more distantly related to *S. telluris* AA8 and *S. hiroshimensis* JCM 4586.

Species delineation among *Streptomyces* spp. was determined using digital DNA–DNA hybridization (dDDH) and average nucleotide identity (ANI) values, with thresholds set at ≥70% for dDDH and ≥95% for ANI. Comparative analysis of dDDH and ANI values for 13 *Streptomyces* strains based on whole-genome alignments revealed that strain ASG80 shares the highest dDDH (86.10%) and ANI (98.22%) values with *S. luteireticuli* JCM 4788, clustering together in the phylogenetic tree (Figure 4B). These findings confirm that strain ASG80 belongs to the same species as *S. luteireticuli* JCM 4788, and we propose naming this strain *Streptomyces luteireticuli* ASG80.

### 3.4. Comparative Genomic Analysis

**Figure 5:**
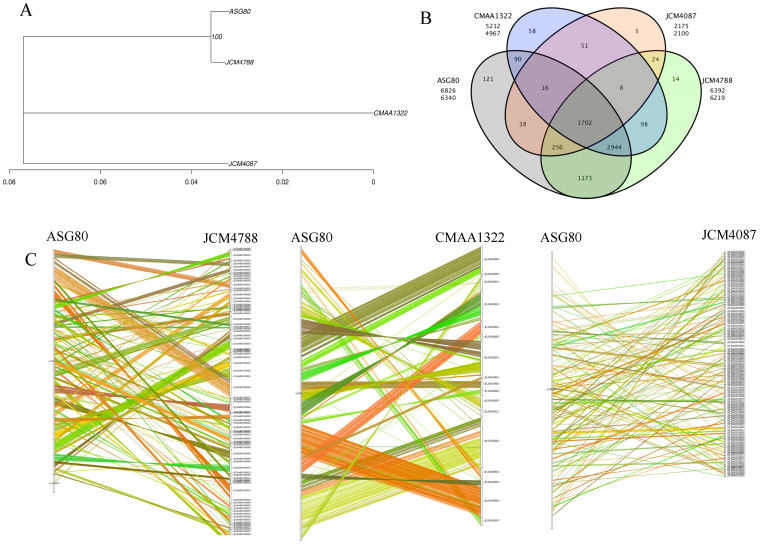
Comparative genomics analysis of strain ASG80. (**A**) Evolutionary relationship between ASG80 and three species. (**B**) Venn diagram of gene family statistics. (**C**) Genome collinearity of ASG80/JCM4788, ASG80/CMAA1322 and ASG80/JCM4087.

Based on the phylogenetic tree and supported by dDDH and ANI values, the genomes of the most closely related strains, *Streptomyces luteireticuli* JCM 4788, *S. thioluteus* JCM 4087, and *S. caatingaensis* CMAA 1322, were selected for comparative genomic analysis with strain ASG80. This analysis was conducted to examine the differences between strain ASG80 and related strains at both intra-species and inter-species levels. Phylogenetic analysis using PhyML software (v3.0) generated an evolutionary tree illustrating the relationships among these species (Figure 5A). The results positioned strain ASG80 and JCM 4788 on the same branch, indicating a close evolutionary relationship. In terms of gene family classification, ASG80 contains 6340 gene families, while strains JCM 4788, JCM 4087, and CMAA 1322 contain 6219, 2100, and 4967 gene families, respectively. Strain ASG80 shares 6093, 2012, and 4752 genes with strains JCM 4788, JCM 4087, and CMAA 1322, respectively. A total of 1702 gene families are conserved across all four strains, with strains ASG80, JCM 4788, JCM 4087, and CMAA 1322 containing 121, 14, 5, and 58 unique genes, respectively (Figure 5B). Further genomic comparisons using Mauve were conducted to align the genomes of strains ASG80, JCM 4788, JCM 4087, and CMAA 1322, revealing multiple rearrangements in syntenic regions among these genomes (Figure 5C).

### 3.5. Broad-Spectrum Antifungal Activity Assay

Increasing evidence indicates that secondary metabolites from *Streptomyces* species can effectively inhibit the growth of *Phytophthora* and other fungal pathogens. To assess the antagonistic activity of strain ASG80 against *Phytophthora* and fungal pathogens, ethyl acetate extracts of strain ASG80 were tested for antifungal activity against 13 different *Phytophthora* and fungal pathogens in vitro (Table 4). Strain ASG80 extract exhibited strong inhibitory effects on the growth of all tested fungal pathogens, with EC_50_ values ranging from 0.8688 to 51.2052 µg/mL. The lowest EC_50_ value was observed against *P. cinnamomi* (0.8688 µg/mL), followed by *P. vignae* (1.3427 µg/mL), *P. nicotianae* (1.6120 µg/mL), *P. sojae* (1.6806 µg/mL), *P. colocasia* (2.1725 µg/mL), *P. melonis* (2.3846 µg/mL), *P. capsici* (2.8159 µg/mL), *P. palmivora* (2.9521 µg/mL), *Magnaporthe* oryzae (8.8389 µg/mL), *Colletotrichum gloeosporioides* (10.5738 µg/mL), *Fusarium graminearum* (13.5908 µg/mL) and *Pestalotiopsis palmarum* (34.6959 µg/mL). In contrast, the highest EC_50_ was observed against *F. oxysporum* (51.2052 µg/mL).

### 3.6. Effect of Strain ASG80 Extract on *Sisal Zebra Disease* Under Greenhouse Conditions

To assess the in vivo inhibitory effect of strain ASG80 extract on *Phytophthora nicotianae*, a pot experiment was conducted under controlled greenhouse conditions. After 30 days of treatment, the disease severity index in the treated groups (ranging from 8.33 to 18.43) was significantly lower than that of the control group (52.45). The disease indices in the T-1 and T-2 treatment groups were reduced by 64.86% and 84.12%, respectively, indicating a positive correlation with the extract concentration (Table 5; Supplementary Figure S2).

## 4. Discussion

In numerous studies, antagonistic *Streptomyces* species have been utilized for plant disease control. For instance, *S. olivoreticuli* ZZ-21 has been effectively utilized against tobacco target spot, caused by *Rhizoctonia solani* [43], while *Streptomyces* sp. Y1-14 has proven effective against banana Fusarium wilt [44]. Additionally, *Streptomyces* species produce diverse secondary metabolites that have been formulated as biopesticides for agricultural applications, including jinggangmycin [45], zhongshengmycin [45], and avermectin [46]. In this study, antagonistic *Streptomyces* strains were screened, leading to the identification of a highly effective strain ASG80. In vivo and in vitro experiments both confirmed the exceptional biocontrol activity exhibited by strain ASG80. Accordingly, the genome of strain ASG80 was sequenced for detailed analysis.

Phylogenetic analysis, informed by whole-genome sequencing, average nucleotide identity (ANI) of 98.22%, and a digital DNA–DNA hybridization (dDDH) value of 86.10%, identified ASG80 as *Streptomyces luteireticuli*. Comparative genomic analysis with *Streptomyces* strains JCM4788, JCM4087, and CMAA1322 revealed that strains ASG80 and JCM4788 belong to the same evolutionary lineage. Gene family clustering indicated the sharing of 1702 genes, with strain ASG80 containing 121 unique genes. Syntenic analysis revealed poor genomic alignment between strain ASG80 and strains JCM4788, JCM4087, and CMAA1322, reflecting numerous insertions, deletions, inversions, and translocations among the genes. These findings imply that strain ASG80 possesses distinctive characteristics, and its biocontrol mechanisms may significantly differ from those observed in other *Streptomyces* strains.

*Streptomyces* species produce a diverse range of secondary metabolites, primarily through the biosynthesis of polyketides, nucleosides, peptides, and hydrolytic enzymes. These metabolites are capable of inhibiting or even eliminating pathogens. In addition, *Streptomyces* generates various bioactive compounds with antimicrobial properties, including enzymes, organic acids, amino acids, immunomodulators, and vitamins [47,48,49]. Whole-genome sequencing enables comprehensive genomic analysis, identification, and the classification of strain ASG80, and facilitates the exploration of potential biosynthetic gene clusters (BGCs) responsible for secondary metabolites. A total of 40 secondary metabolite biosynthesis gene clusters were identified in the genome of strain ASG80. Among them, 11 clusters exhibited significant similarity (greater than 50%) to known BGCs, with four clusters—antipain, geosmin, diisonitrile antibiotic SF2768, and aureothin—demonstrating 100% similarity. Antipain is a novel analogue that inhibits pain by suppressing PAR signaling via protease inhibition, subsequently reducing excitatory neuropeptide release [50]. Diisonitrile antibiotic SF2768 acts as a copper carrier by binding specifically to copper [51], while aureothin exhibits a range of biological activities, including antifungal [52,53], antiviral [54], antitumor [53], nematicidal [55], and larvicidal effects [56]. Of note, a significant number of BGCs exhibited minimal similarity, with similarity scores below 50%, and many others under 20%. Six BGCs displayed no detectable similarity to known clusters, suggesting that strain ASG80 harbors numerous genes with potentially novel functions, thereby highlighting its significant research potential.

In recent years, an increasing number of *Streptomyces* species have been identified as possessing anti-oomycete activity, positioning them as potential biocontrol agents against *Phytophthora* diseases. For example, *Streptomyces* sp. FXP04 and *Streptomyces* sp. A2-16 were demonstrated to control late blight in potatoes [57,58], while *S. rochei* IT20 and *S. vinaceusdrappus* SS14 effectively inhibit *Phytophthora* in chili peppers [59]. However, compared to bacterial and fungal antagonists, relatively few studies report on the use of *Streptomyces* for *Phytophthora* biocontrol. In this study, strain ASG80 and its extracts showed potent inhibitory effects against both oomycete and fungal pathogens. The extracts exhibited EC_50_ values between 0.8 and 3.0 µg/mL against oomycete pathogens, whereas EC_50_ values exceeded 8.0 µg/mL for fungal pathogens, indicating a stronger inhibitory effect on oomycetes.

Sisal zebra disease, induced by *Phytophthora nicotianae*, represents a significant threat to sisal cultivation. Chemical fungicides are commonly utilized to manage this pathogen. In an effort to minimize reliance on synthetic fungicides, recent research has increasingly centered on identifying effective biological control agents, such as *Bacillus* [60], *Pseudomonas* [61], *Trichoderma* [62], and yeast strains [63], that demonstrate efficacy in inhibiting *P. nicotianae*. In this study, strain ASG80 extracts exhibited broad-spectrum in vitro anti-*Phytophthora* activity and substantially decreased zebra stripe disease incidence in sisal in vivo, demonstrating a control effect comparable to that of metalaxyl. The observed reduction in disease incidence was concentration-dependent, suggesting that the extracts function as the principal inhibitory component. Additionally, strain ASG80 displayed broad-spectrum activity against *Phytophthora* and fungal pathogens, exhibiting significant inhibitory effects on 13 different pathogens. This finding aligns with previous reports indicating that promising biocontrol strains frequently exhibit a broad antimicrobial spectrum [64]. To the best of our knowledge, this study represents the first investigation into the biocontrol potential of *Streptomyces luteireticuli* against *Phytophthora* species. Strain ASG80 demonstrates significant potential as a biocontrol agent for the management of *Phytophthora* diseases.

## 5. Conclusions

In this study, the complete genomic profile of strain ASG80 was comprehensively analyzed, and the functional roles of its associated genes were investigated. The findings provide insights that could support further research into the regulatory mechanisms of active secondary metabolite biosynthesis in strain ASG80. Furthermore, strain ASG80 extracts exhibited broad-spectrum in vitro anti-*Phytophthora* activity and significantly reduced the incidence of sisal zebra disease in vivo. These results indicate that strain ASG80 possesses considerable potential as a biocontrol agent against *Phytophthora* diseases in plants. The development and application of strain ASG80 could contribute significantly to green control strategies for plant diseases, providing a valuable resource for utilizing antagonistic *Streptomyces* in biocontrol. Additionally, this strain offers a foundation for the experimental exploration and application of novel antibacterial compounds.

## Figures and Tables

**Table 1:** The inhibition rate (%) of the pathogens by strain ASG80.

Pathogens	Mycelial Inhibition (%)
*Phytophthora colocasiae*	83.74 ± 0
*Phytophthora cinnamomi*	72.87 ± 0.46
*Phytophthora palmivora*	75.63 ± 0.27
*Phytophthora capsici*	61.27 ± 0.18
*Phytophthora vignae*	88.43 ± 0.31
*Phytophthora sojae*	56.70 ± 0.26
*Phytophthora melonis*	70.53 ± 0.15
*Colletotrichum gloeosporioides*	62.78 ± 0.60
*Magnaporthe oryzae*	85.87 ± 0
*Fusarium oxysporum*	43.21 ± 0.16
*Fusarium graminearum*	44.96 ± 0.31
*Pestalotiopsis palmarum*	58.95 ± 0.16

**Table 2:** Genome characteristics of strain ASG80.

Database	Number of Annotated Functional Proteins	Proportion/%
eggNOG	5549	70.54
GO	4598	58.45
KEGG	2385	30.32
Nr	7531	95.73
Pfam	5793	73.64
Swiss-prot	3440	43.73
TrEMB	7510	95.46
All databases	7549	95.96

**Table 3:** Secondary metabolite clusters in strain ASG80 predicted by antiSMASH.

Region	Type	From	To	Most Similar Known Cluster	Similarity
Clusters 1	Terpene, NRPS, NRPS-like	60,185	117,028	antipain	100%
Clusters 2	T1PKS	169,015	216,487	himastatin	8%
Clusters 3	T1PKS	242,821	288,100	petrichorin	5%
Clusters 4	NRPS, T1PKS	473,674	553,694	tallysomycin	55%
Clusters 5	Terpene	578,046	600,082	tylactone	6%
Clusters 6	Terpene	610,891	631,859	-	
Clusters 7	Lassopeptide, T1PKS	638,187	696,947	ulleungdin	75%
Clusters 8	Thiopeptide	748,305	808,571	geosmin	100%
Clusters 9	T1PKS, NRPS	816,029	911,078	peucechelin	20%
Clusters 10	T1PKS, NRPS-like	930,265	1,105,264	conglobatin	26%
Clusters 11	NRPS	1,122,162	1,166,496	diisonitrile antibiotic SF2768	100%
Clusters 12	T1PKS, phenazine	1,194,939	1,257,441	aureothin	100%
Clusters 13	NRPS, T1PKS	1,295,327	1,467,303	sceliphrolactam	48%
Clusters 14	NRPS-like, arylpolyene	1,547,967	1,590,881	lankacidin C	20%
Clusters 15	RiPP-like	1,632,931	1,644,841	-	
Clusters 16	Lasso peptide	1,746,532	1,769,080	lagmysin	80%
Clusters 17	Terpene	1,779,551	1,800,171	-	
Clusters 18	T1PKS	1,890,303	1,957,177	cremimycin	10%
Clusters 19	NI-siderophore	2,265,382	2,295,133	-	
Clusters 20	Terpene, NRPS	2,725,486	2,770,746	pyrroloformamide	37%
Clusters 21	Thioamide-NRP, T1PKS	3,661,290	3,719,169	5-isoprenylindole-3-carboxylate β-D-glycosyl ester	33%
Clusters 22	Butyrolactone, T3PKS	4,656,145	4,697,411	neocarzinostatin	8%
Clusters 23	Terpene	4,924,188	4,945,345	ebelactone	5%
Clusters 24	T3PKS	5,734,254	5,775,303	violapyrone B	28%
Clusters 25	NI-siderophore	6,121,786	6,151,618	kinamycin	19%
Clusters 26	RiPP-like	6,210,115	6,220,963	-	
Clusters 27	T3PKS, phenazine	6,247,692	6,295,310	endophenazine	47%
Clusters 28	T2PKS	6,367,291	6,439,815	allocyclinone	43%
Clusters 29	Phenazine, NRPS	6,617,890	6,676,804	streptophenazine	24%
Clusters 30	Indole	6,688,763	6,712,068	AT2433-A1	14%
Clusters 31	NAPAA, terpene	6,721,859	6,774,236	hopene	76%
Clusters 32	NRPS-like	6,831,419	6,873,260	kitacinnamycin	7%
Clusters 33	NRPS-like	6,898,154	6,961,149	cyphomycin	5%
Clusters 34	Other, terpene	6,985,496	7,026,419	ECO-0501	4%
Clusters 35	Other	7,182,203	7,223,099	griseusin	15%
Clusters 36	RiPP-like	7,298,812	7,310,096	-	
Clusters 37	NRPS, T1PKS, terpene	7,364,647	7,465,253	hexacosalactone A	18%
Clusters 38	NRPS-like, CDPS	7,500,628	7,543,702	guanipiperazine	80%
Clusters 39	T1PKS, NRPS	7,647,760	7,783,943	qinichelins	83%
Clusters 40	T3PKS, T1PKS, furan	7,816,344	7,894,681	paerucumarin	60%

**Table 4:** Effects of strain ASG80 extracts on mycelial growth of phytopathogens.

Pathogens	Regression Equation	EC_50_ (μg/mL)	Correlation Coefficient
*Phytophthora vignae*	y = 1.3297x + 4.8299	1.3427	0.9864
*Phytophthora sojae*	y = 1.6772x + 4.6219	1.6806	0.9985
*Phytophthora palmivora*	y = 1.7603x + 4.1724	2.9521	0.9920
*Phytophthora nicotiana*	y = 0.8279x + 4.8283	1.6120	0.9952
*Phytophthora melonis*	y = 1.1621x + 4.5614	2.3846	0.9994
*Phytophthora colocasiae*	y = 1.7260x + 4.4184	2.1725	0.9687
*Phytophthora cinnamomi*	y = 1.6358x + 5.0999	0.8688	0.9998
*Phytophthora capsici*	y = 1.7841x + 4.1978	2.8159	0.9933
*Pestalotiopsis palmarum*	y = 1.3937x + 2.8532	34.6959	0.9895
*Magnaporthe oryzae*	y = 1.1531x + 3.9087	8.8389	0.9807
*Fusarium oxysporum*	y = 1.1694x + 3.0012	51.2052	0.9956
*Fusarium graminearum*	y = 1.5994x + 3.1874	13.5908	0.9958
*Colletotrichum gloeosporioides*	y = 1.7642x + 3.1930	10.5738	0.9938

**Table 5:** The inhibitory effect of strain ASG80 extract on sisal zebra disease.

Treatment	Incidence Rate (%)	Disease Index	Control Effect (%)
CK	0	/	/
Pn	100.00	52.45	/
T-1	30.00	8.33	84.12
T-2	46.67	18.43	64.86
Me-2	36.67	11.99	77.14

## Data Availability

Sequence data from this article can be found in GenBank at https://www.ncbi.nlm.nih.gov/datasets/genome/ (accessed on 4 August 2020) with the accession numbers listed in the Results Section. All other relevant data are within the paper.

## Supplementary Materials

The following supporting information can be downloaded at: https://www.mdpi.com/article/10.3390/microorganisms12112255/s1, Figure S1. A broad-spectrum antifungal activity of strain ASG80 against the selected twelve phytopathogenic. Figure S2. Effects of strain ASG80 extract on zebra stripe disease in potted plants. (A) No *Phytophthora nicotianae*, sterile water(CK). (B) *P. nicotianae*, sterile water. (C) *P. nicotianae*, ASG80 extract at a 1:1000 dilution. (D) *P. nicotianae*, ASG80 extract at a 1:2000 dilution. (E) *P. nicotianae*, metalaxyl at a 1:2000 dilution.
