# Supplementary material for: Whole Genome Sequencing and Biocontrol Potential of Streptomyces luteireticuli ASG80 Against Phytophthora Diseases
Source: Microorganisms. 2024 Nov 7;12(11):2255. doi: 10.3390/microorganisms12112255 (PMC11596116; doi:10.3390/microorganisms12112255)
Supplement: Supplementary file 1 [file microorganisms-12-02255-s001.zip › microorganisms-3293017-supplementary.pdf]

# Supplementary material

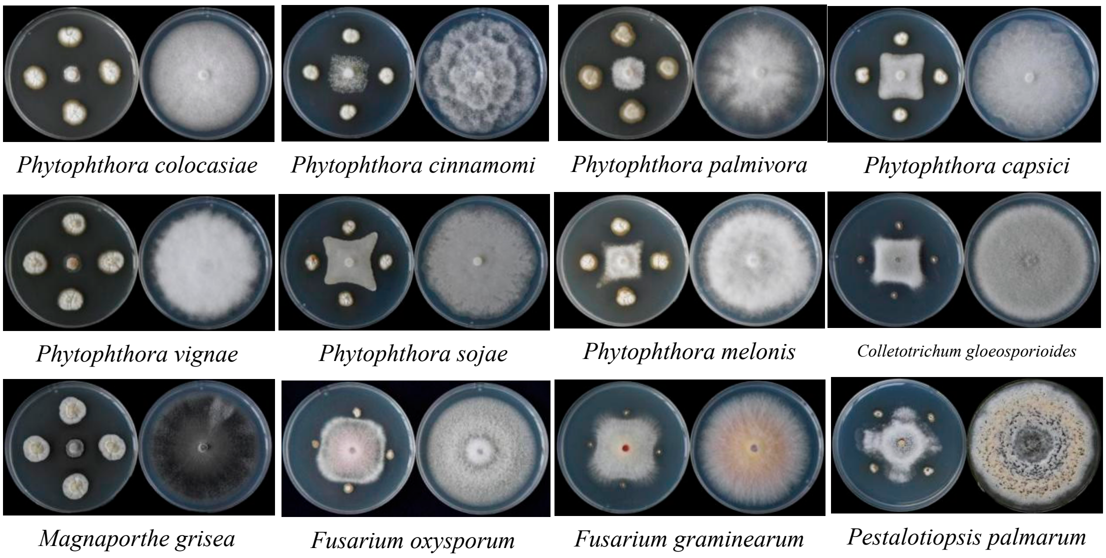


**Figure S1.** A broad-spectrum antifungal activity of strain ASG80 against the selected twelve phytopathogenic.

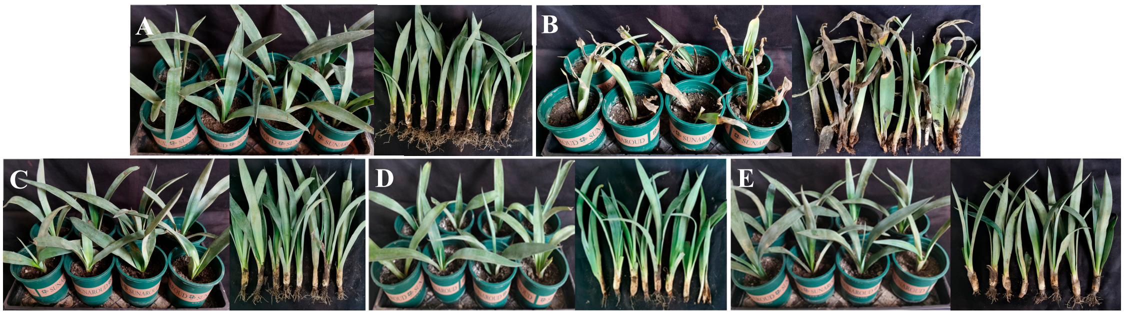


**Figure S2.** Effects of strain ASG80 extract on zebra stripe disease in potted plants. (A) No *Phytophthora nicotianae*, sterile water(CK). (B) *P. nicotianae*, sterile water. (C) *P. nicotianae*, ASG80 extract at a 1:1000 dilution. (D) *P. nicotianae*, ASG80 extract at a 1:2000 dilution. (E) *P. nicotianae*, metalaxyl at a 1:2000 dilution.
